# Perineural Dexamethasone Versus Intravenous Dexamethasone as an Adjunct to Local Anaesthetic Mixture in Supraclavicular Brachial Plexus Block: A Prospective Double-Blind Randomized Controlled Study

**DOI:** 10.7759/cureus.107346

**Published:** 2026-04-19

**Authors:** Leenu G Ninan, Sivaramakrishnan Dhamotharan, Amoolya Kamalnath, Sivakumar Segaran

**Affiliations:** 1 Anaesthesiology, Kanad Hospital, Al Ain, ARE; 2 Anaesthesiology and Critical Care, Indira Gandhi Medical College, Pondicherry, IND; 3 Anaesthesiology, Pondicherry Institute of Medical Sciences, Pondicherry, IND

**Keywords:** intravenous dexamethasone, motor blockade, perineural dexamethasone, regional anaesthesia, sensory blockade, supraclavicular brachial plexus block

## Abstract

Introduction

Supraclavicular brachial plexus block is most preferred for upper limb surgeries since it produces conduction blockade of the trunks of the brachial plexus. Additives are used with local anaesthetics to enhance the effect of analgesia in patients. Therefore, this study is aimed at comparing the efficacy, onset, and duration of block, intraoperative hemodynamics, and postoperative complications, if any, when dexamethasone is used as an adjuvant in supraclavicular block perineurally and intravenously.

Methods

Ninety adult patients scheduled for elective forearm and hand surgery were randomly divided into three groups, group P, group I, and group S, which received perineural dexamethasone, intravenous dexamethasone, and normal saline, respectively. Supraclavicular brachial plexus block was administered. Hemodynamic parameters were recorded. Assessment of sensory and motor block was done till complete sensory blockade was achieved. Hemodynamic parameters and onset and duration of sensory and motor block were compared using a one-way analysis of variance (ANOVA) test. A p-value of <0.05 was considered statistically significant.

Results

Onset of sensory and motor blockade was statistically significantly faster after administering perineural dexamethasone compared to intravenous dexamethasone (p=0.003 and p=0.001) and the control group (p<0.001), respectively. The duration of sensory and motor blockade was statistically significantly longer after perineural dexamethasone compared to intravenous dexamethasone (p<0.001) and the control group (p<0.001), respectively. There were no statistically significant hemodynamic changes among the three groups intraoperatively.

Conclusion

Dexamethasone as an adjuvant to a local anaesthetic mixture in a supraclavicular block speeds the onset and prolongs the duration of sensory and motor blockade compared to intravenous dexamethasone.

## Introduction

Regional anaesthesia (RA) provides effective analgesia for surgeries of the upper limbs; thereby, exposures to multiple drugs, airway instrumentation, and hence hemodynamic pressor response and postoperative sore throat [1] that may occur during general anaesthesia (GA) are avoided. The advantage of a peripheral nerve block (PNB) is anesthetizing limited parts of the body while preserving patients' consciousness. PNBs using ultrasound guidance are preferred due to better accuracy and efficacy when compared to using a peripheral nerve stimulator and over landmark techniques (which have a higher failure rate). Ultrasound guidance enables the real-time visualization of the anatomy, needle trajectory, and perineural local anaesthetic (LA) spread, reducing LA requirement and hence reducing LA systemic toxicity risk [2]. Brachial plexus block, the most commonly performed PNB, is used solely or alongside GA, intraoperatively, to reduce the general anaesthetic agent requirement as well as for postoperative analgesia. The advantage of the brachial plexus block is that it provides sufficient muscle relaxation and superior analgesia. Among the different methods of brachial plexus blocks, the supraclavicular block (SCB) is the most favoured and highly successful route, due to likely conduction blockade at the distal trunks of the brachial plexus [3]. Short-acting and quick-onset (lignocaine) and long-acting and slow-onset (bupivacaine and ropivacaine) LAs have been used in brachial plexus blockade. When combined, a faster onset and prolonged action is observed. Adrenaline (when mixed with lignocaine), by producing vasoconstriction, reduces the absorption of LA. Many additives are used along with LAs during SCB to increase the analgesic effect in patients, a few being neostigmine, clonidine, tramadol, dexamethasone, dexmedetomidine, etc. [4,5]. An ideal drug for PNBs with hastened onset and enhanced duration of LA providing excellent hemodynamic stability with almost no complications is yet to be elucidated. Though the mechanism of dexamethasone's action is not fully understood when used locally, it is being utilized for pain management. Dexamethasone inhibits the production of pro-inflammatory cytokines (e.g., tumour necrosis factor alpha, interleukin-1 beta) and stabilizes lysosomal membranes, thus helping in suppressing inflammation and reducing pain. It inhibits potassium channels in vascular smooth muscle cells causing depolarization and vasoconstriction, thus prolonging the duration of action of a LA injected along with it. Also, the duration of analgesia has been shown to be enhanced when administered perineurally along with LAs [6]. Intravenous (IV) dexamethasone also helps in reducing the postoperative analgesic requirement [7]. Hence, by comparing the IV route with the perineural route of administration of dexamethasone, we can assess if dexamethasone given perineurally would be more efficacious and produce a more optimal surgical environment.

Our aim was to assess the efficacy of dexamethasone by assessing the onset and duration of sensory and motor blockade when added as an adjunct to LA mixture in SCB and compare it when given perineurally and IV. Our primary objective was to compare the postoperative duration of sensory and motor blockade between patients receiving perineural dexamethasone (Pd), intravenous dexamethasone (IVd), and a placebo (control), having been grouped as group P, group I, and group S, respectively. Our secondary objectives were to (i) compare the onset of the block among the groups, (ii) compare the hemodynamic parameters among the study groups, and (iii) assess any intraoperative and postoperative complications. Our research hypothesis was that the addition of dexamethasone as an adjuvant perineurally hastens the onset and prolongs the duration of the supraclavicular brachial plexus block rather than when given intravenously.

## Materials and methods

A prospective double-blind randomized controlled study was conducted at Pondicherry Institute of Medical Sciences and designed after obtaining clearance from the institute's Institutional Ethics Committee on October 5, 2015 (approval number: RC/15/15). The study was also registered in the Clinical Trials Registry-India (CTRI) (CTRI registration number: REF/2016/05/011445). The period of study was for a duration of two years from October 2015 to April 2017.

Patients from the ages of 18 to 60 years, belonging to the American Society of Anesthesiologists (ASA) physical statuses I and II and who underwent elective upper limb surgeries (forearm and hand), were included in the study. Pregnant women; patients who developed hypersensitivity to LA agents; those with diabetes mellitus, pre-existing peripheral neuropathy, and neuro-psychiatric disorder; patients on psychotropic drugs for a prolonged period; those having bleeding disorders or deranged coagulation profile; patients on anti-coagulant therapy and prolonged steroid therapy; and those having clavicular fractures with/without chest/shoulder deformities on the operating side were excluded from the study. Patients who met the inclusion criteria were assessed preoperatively and were informed about the study.

Routine investigations revealed baseline values. For patients above 45 years of age, chest X-ray and electrocardiography were also done. After obtaining written informed consent, all patients were given pre-medications of 1 mg tablet of lorazepam and tablet ranitidine 150 mg at bedtime and at six a.m. orally with sips of water. An 18G IV cannula was secured on the contralateral upper limb. After the patients were shifted to the operating table, ASA standard monitors were connected along with oxygen at 5 L/min by a simple face mask.

Ninety patients of both genders were randomly allocated (using computer-generated random numbers, with a block size of nine patients in each block of a total of 10 blocks) into three groups, group P, group I, and group S. A qualified, well-trained anaesthesiologist who was blinded to the study performed the procedure on the patient who was also blinded to the study (the drug being used, dexamethasone or normal saline (NS), was pre-loaded in a sterile manner by another senior anaesthesiologist and was not revealed to either the anaesthesiologist performing the procedure or the patient). The anaesthesiologist who was blinded to the drug and performed the procedure followed up and assessed the patient postoperatively. Under aseptic precautions, the brachial plexus was identified using an ultrasound image-guided (SonoSite M-Turbo 13-6 MHz high-frequency linear probe (HFL 38) (FUJIFILM SonoSite Inc., Bothell, WA, USA)) supraclavicular approach. Once the brachial plexus was identified and the location of the needle tip was confirmed by hydrodissection, the drug was deposited within the brachial plexus sheath. Patients in group P received 18 ml of 0.5% bupivacaine, 7 ml of 2% lignocaine with adrenaline, and 8 mg (2 ml) of dexamethasone perineurally and an IV infusion of 100 ml of NS. Patients in group I received 18 ml of 0.5% bupivacaine, 7 ml of 2% lignocaine with adrenaline, and 2 ml of NS perineurally and 8 mg of dexamethasone IV in 100 ml of NS. Patients in group S received 18 ml of 0.5% bupivacaine, 7 ml of 2% lignocaine with adrenaline, and 2 ml of NS perineurally and an IV infusion of 100 ml of NS.

The patients' heart rate (HR), blood pressure (BP), and oxygen saturation were recorded: at baseline, pre-procedure, after performing the block, and every five minutes for the initial half an hour and every 10 minutes till the end of surgery. By checking for response to pin-prick with a 23-G hypodermic needle, the sensory block was assessed. After the drug was injected, sensory blockade was assessed at five-minute intervals in the dermatomal areas corresponding to the median, radial, ulnar, and musculocutaneous nerves until complete sensory blockade was achieved. Along the distribution of any of the above-mentioned nerves, a dull sensation to pin-prick heralded the sensory block onset. Complete loss of sensation to pin-prick ensured a complete sensory block. Sensory block grades are the following: Grade 0 (sharp pin felt), Grade 1 (analgesia, dull sensation felt), and Grade 2 (anaesthesia, no sensation felt).

After drug injection, assessment of motor block was carried out by the same observer at five-minute intervals till complete motor blockade. Grade 1 was the onset of motor blockade, and Grade 2 was complete motor block. Motor block grading is as follows: Grade 0 (full flexion and extension of the elbow, wrist, and fingers denotes normal motor function), Grade 1 (ability to move the fingers only denotes decreased motor strength), and Grade 2 (inability to move the fingers denotes complete motor block).

Even 30 minutes after drug injection, when the segments supplied by any of the above-mentioned nerves did not exhibit analgesia, the block was considered incomplete. Based on the body weight and calculation of the maximum dose of lignocaine that can be given to the patient, 5-10 ml of 2% lignocaine was administered locally in these cases. If the patient complained of pain even after local infiltration, injection fentanyl 1 µg/kg was used as a rescue analgesic, and these cases were included in the study. A failed block was defined as more than one nerve remaining unaffected. In these cases, GA was given intraoperatively, and these cases were excluded from the analysis.

Based on the time from the onset of loss of sensation as well as the fingers' movement till the patient complained of pain (visual analog scale >3) and return of finger movements, the duration of the sensory and motor block, respectively, was assessed. Any complications were looked for and noted intraoperatively and 24 hours postoperatively.

Statistical analysis

Descriptive statistics such as mean and standard deviation were used for continuous variables and number and percentage for categorical variables. One-way analysis of variance (ANOVA) test was used for age, chi-squared test for gender and ASA status, and ANOVA for body weight. By using a one-way ANOVA test, the onset and duration of sensory and motor blockade were determined. Further, a Dunnett post-hoc test was also done, and a p-value of <0.05 was considered statistically significant. By applying Yates' corrected chi-squared test, additional drug supplementation was analysed. Hemodynamic parameters (HR, systolic blood pressure (SBP), diastolic blood pressure (DBP), mean arterial pressure (MAP)) were compared using a one-way ANOVA test over time intervals. The software used was IBM SPSS Statistics for Windows, V. 26.0 (IBM Corp., Armonk, NY, USA).

**Figure 1:**
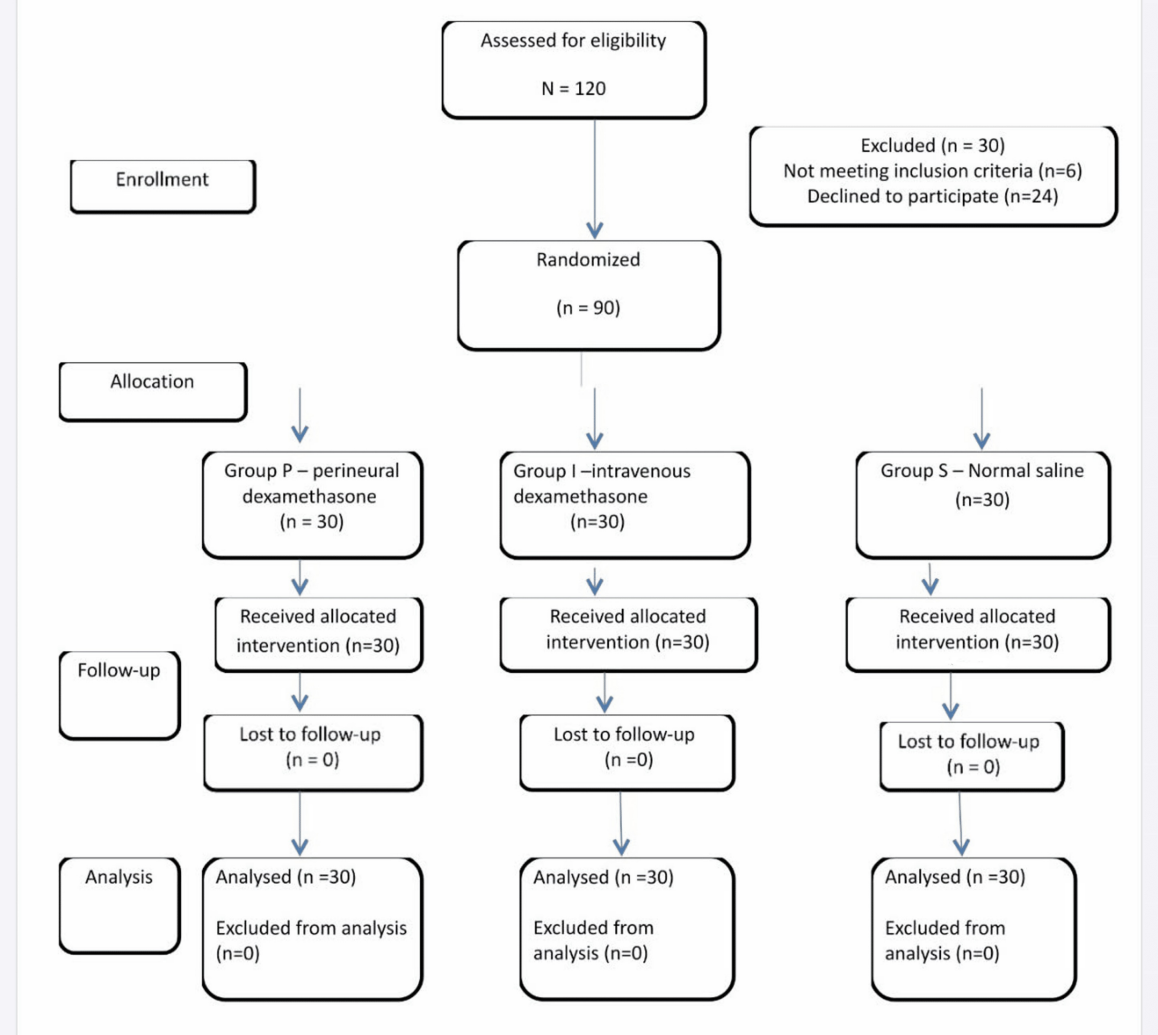
CONSORT diagram CONSORT: Consolidated Standards of Reporting Trials

The sample size for our study has been calculated using the onset of sensory blockade. Kumar et al. showed that the onset time of sensory blockade was 18.26±1.25 minutes when patients received 38 ml of 0.25% bupivacaine with 2 ml (8 mg) of dexamethasone [8]. To demonstrate that the addition of 8 mg of dexamethasone to 27 ml of LA mixture (18 ml of 0.5% bupivacaine and 7 ml of 2% lignocaine with adrenaline) would reduce the onset time of sensory blockade by 6%, and considering an 80% power and a 0.05 alpha error, the sample size required per group in our study was calculated with the help of nMaster (Informer Technologies, Inc., Los Angeles, CA, USA) to be 25. Assuming a dropout rate of 10%, a sample size of 28 was calculated for each group. However, a final sample size of 90 with 30 patients in each group was considered to be studied (Figure 1).

## Results

The age, gender, ASA status, and body weight were comparable between the three groups, and there was no statistically significant difference between the three groups (Table 1).

**Table 1:** Demographic characteristics of the participants Gender and ASA grade are represented as numbers (n) and percentages (%). Age and body weight are represented as mean and SD. One-way ANOVA test was used for age, chi-squared test was used for gender and ASA status, and ANOVA was used for body weight. A p-value of <0.05 is considered significant. ASA: American Society of Anesthesiologists: SD: standard deviation; ANOVA: analysis of variance

Demographic parameters	Group	Control	Intravenous dexamethasone	Perineural dexamethasone	P-value
Gender (n, %)	Male	27 (37.0)	24 (32.9)	22 (30.1)	0.252
	Female	3 (17.7)	6 (35.3)	8 (47.1)	
ASA (n, %)	I	21 (34.4)	22 (36.1)	18 (29.5)	0.516
	II	9 (31.0)	8 (27.6)	12 (41.4)	
Age (mean (SD))	-	35.63 (11.294)	33.60 (9.912)	39.77 (13.242)	0.115
Body weight (mean (SD))	-	67.17 (9.959)	67.67 (10.226)	67.03 (10.060)	0.968

For age, a one-way ANOVA test was used. For gender and ASA status, the chi-squared test was used, and for body weight, ANOVA was used.

**Figure 2:**
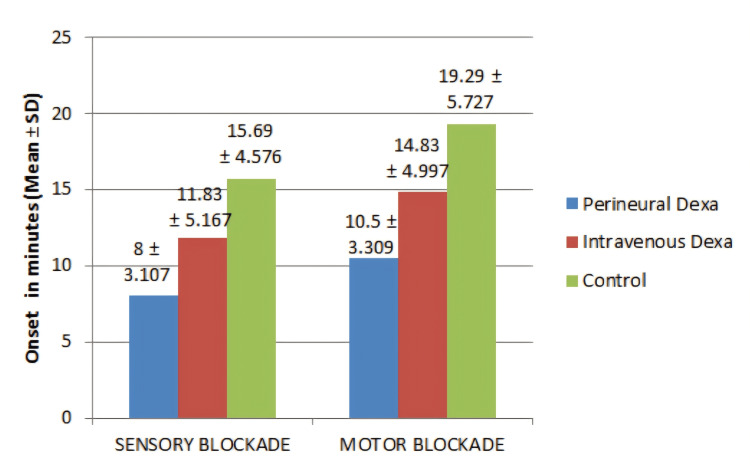
Comparison of the onset of sensory and motor block among the three groups The onset (in minutes) of sensory and motor blockade is denoted in the graph by mean±SD. SD: standard deviation; Dexa: dexamethasone

As determined by one-way ANOVA, in the onset of sensory and motor blockade, there was a statistically significant difference between the three groups. Onset of blockade, both sensory and motor, was faster with Pd when compared to IVd and the control group (Figure 2).

A Dunnett post-hoc test revealed that the onset of blockade (sensory and motor) was statistically significantly faster after Pd (8.00±3.107 minutes and 10.50±3.309 minutes) compared to IVd (11.83±5.167 minutes (p=0.003) and 14.83±4.997 minutes (p=0.001)) and the control group (15.69±4.576 minutes (p<0.001) and 19.29±5.727 minutes (p<0.001)). Similarly, the onset of blockade (sensory and motor) was statistically significantly faster after IVd (11.83±5.167 minutes and 14.83±4.997 minutes) compared to the control group (15.69±4.576 minutes (p=0.011) and 19.29±5.727 minutes (p=0.008)) (Table 2).

**Table 2:** Onset of sensory blockade (in minutes) and motor blockade (in minutes) in all three groups *The mean difference is significant at the 0.05 level. A Dunnett post-hoc test was used to calculate the p-value for pairwise comparison among the three groups. A p-value of <0.05 was considered significant.

(I) Group	(J) Group	Mean difference (I-J)	Standard error	P-value	95% confidence interval	
					Lower bound	Upper bound
Onset of sensory blockade (minutes)						
Perineural dexamethasone	Intravenous dexamethasone	-3.833^*^	1.101	0.003	-6.55	-1.11
Control	Perineural dexamethasone	7.690^*^	1.022	0.000	5.17	10.21
Intravenous dexamethasone	Control	-3.856^*^	1.270	0.011	-6.98	-0.74
Onset of motor blockade (minutes)						
Perineural dexamethasone	Intravenous dexamethasone	-4.333^*^	1.094	0.001	-7.03	-1.63
Control	Perineural dexamethasone	8.786^*^	1.240	0.000	5.71	11.86
Intravenous dexamethasone	Control	-4.452^*^	1.416	0.008	-7.94	-0.97

**Figure 3:**
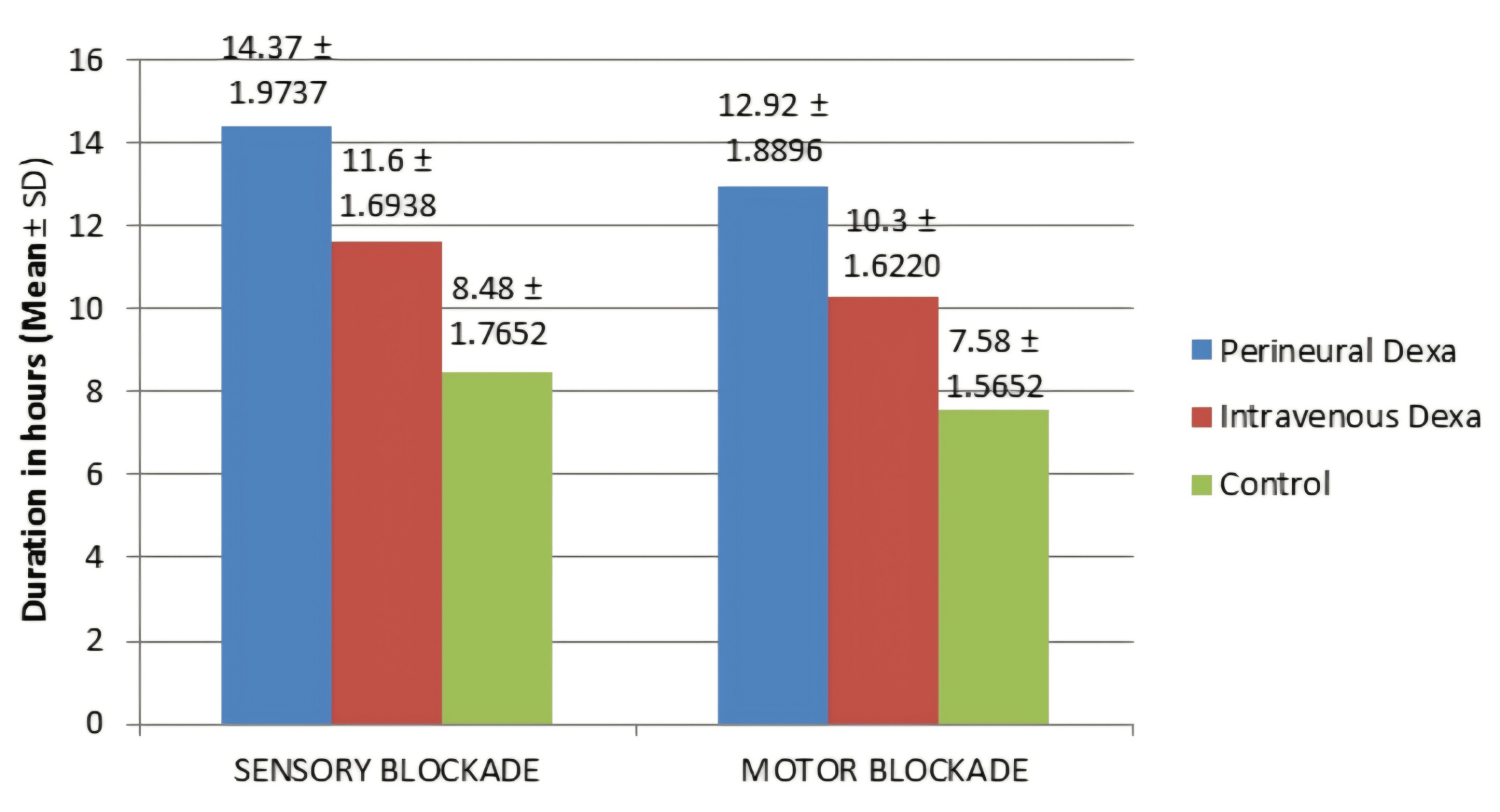
Comparison of the duration of sensory and motor block among the three groups The duration (in hours) of sensory and motor blockade is denoted in the graph by mean±SD. SD: standard deviation; Dexa: dexamethasone

There was a statistically significant difference between the three groups in the duration of sensory and motor blockade as deduced by one-way ANOVA. The sensory and motor blockade were prolonged in the perineural group when compared to the IV and control groups (Figure 3).

A Dunnett post-hoc test revealed that the duration of sensory and motor blockade was statistically significantly longer after Pd (14.37±1.974 hours and 12.92±1.89 hours) compared to IVd (11.60±1.694 hours (p<0.001) and 10.30±1.622 hours (p<0.001)) and the control group (8.48±1.765 hours (p<0.001) and 7.58±1.57 hours (p<0.001)). Similarly, the duration of blockade (sensory and motor) was statistically significantly longer after IVd (11.60±1.694 hours and 10.30±1.622 hours; p<0.001) compared to the control group (8.48±1.765 hours (p<0.001) and 7.58±1.57 hours (p<0.001)) (Table 3).

**Table 3:** Duration of sensory blockade (in hours) and motor blockade (in hours) in all three groups *The mean difference is significant at the 0.05 level. A Dunnett post-hoc test was used to calculate the p-value for pairwise comparison among the three groups. A p-value of <0.05 was considered significant.

(I) Group	(J) Group	Mean difference (I-J)	Standard error	P-value	95% confidence interval	
					Lower bound	Upper bound
Duration of sensory blockade (hours)						
Perineural dexamethasone	Intravenous dexamethasone	2.7667^*^	0.4748	0.000	1.600	3.934
Control	Perineural dexamethasone	-5.8839^*^	0.4871	0.000	-7.081	-4.687
Intravenous dexamethasone	Control	3.1172^*^	0.4506	0.000	2.010	4.225
Duration of motor blockade (hours)						
Perineural dexamethasone	Intravenous dexamethasone	2.6167^*^	0.4547	0.000	1.499	3.734
Control	Perineural dexamethasone	-5.3333^*^	0.4480	0.000	-6.435	-4.232
Intravenous dexamethasone	Control	2.7167^*^	0.4115	0.000	1.706	3.727

Additional drug supplementation was required in one SCB, respectively, where Pd (3.3%) and IVd (3.3%) were used, and in two SCBs (6.7%) where NS was used. Applying Yates' corrected chi-squared test, this was found to be not statistically significant (p=0.856). There were no complications in the perioperative period.

**Figure 4:**
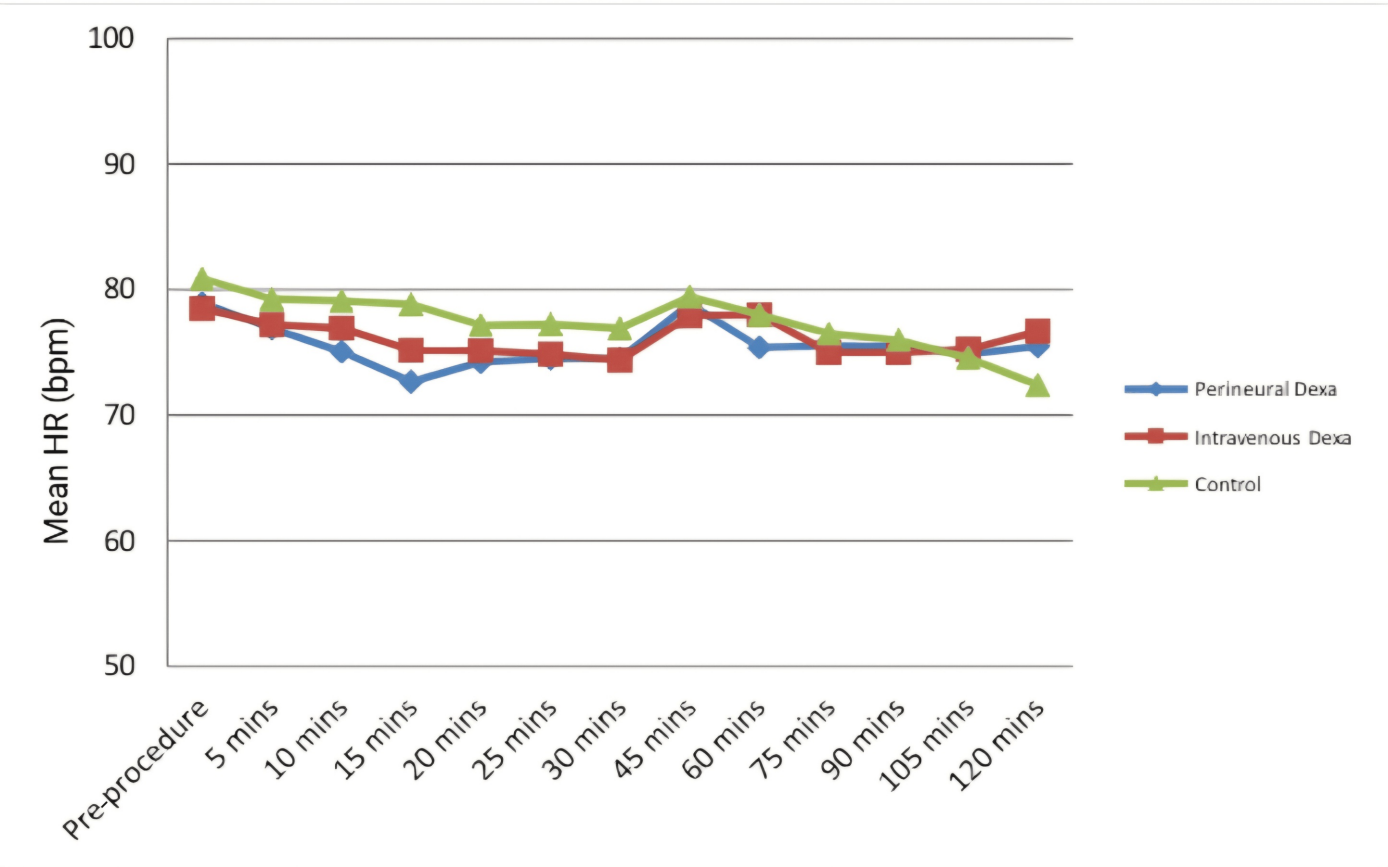
Changes in the mean HR (in bpm) among the three groups HR: heart rate; bpm: beats per minute; Dexa: dexamethasone

**Figure 5:**
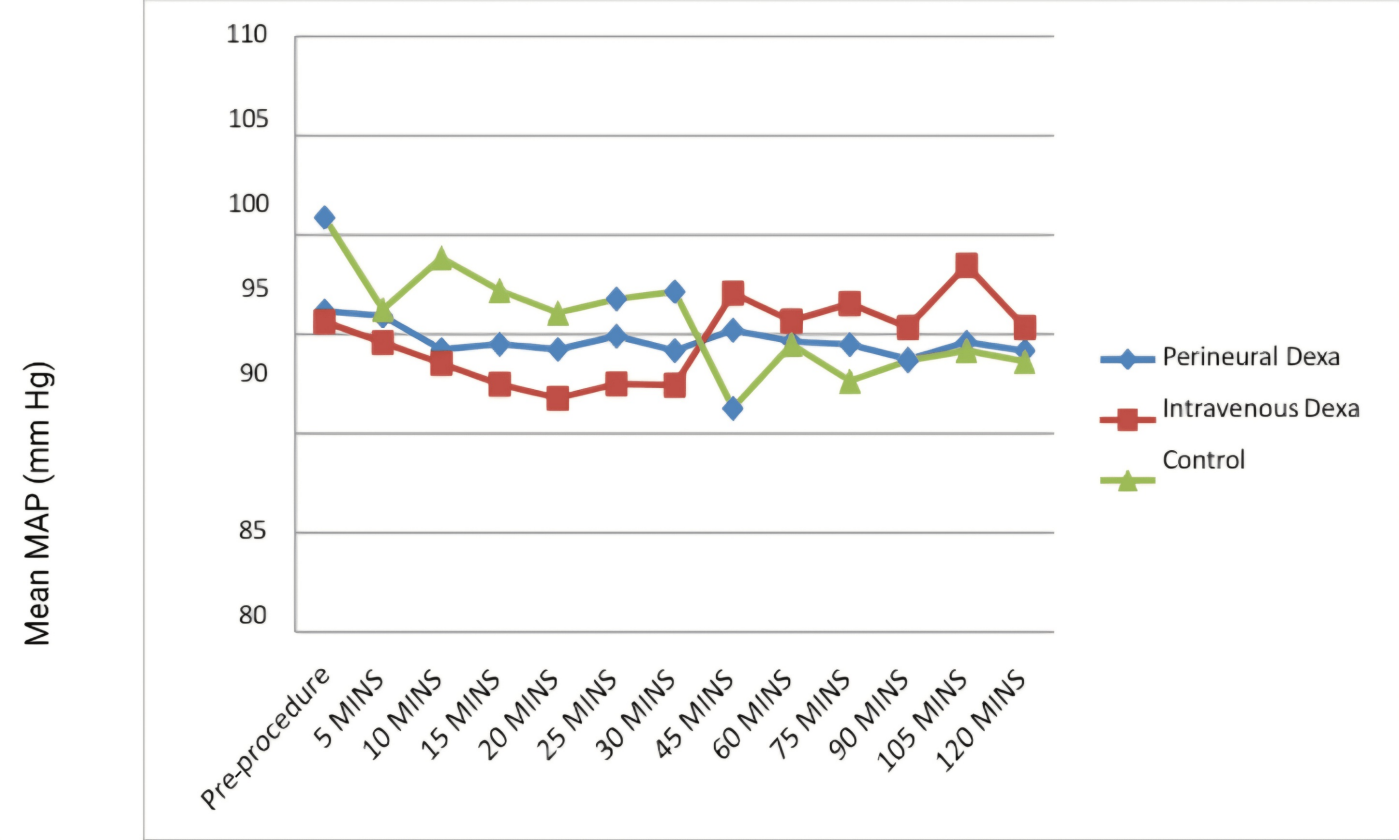
Changes in the mean MAP (in mm Hg) among the three groups MAP: mean arterial pressure; Dexa: dexamethasone

Using a one-way ANOVA test, there was no statistically significant difference in HR (Figure 4) and SBP between all three groups. After applying a one-way ANOVA test, there was no statistically significant difference in DBP and MAP (Figure 5) between all three groups except at 15 minutes (p=0.165; F-value-3.326) and 10 minutes (p=0.025; F-value-4.009), respectively, which were clinically not significant.

## Discussion

RA has become the choice of anaesthesiologists, especially for patients with comorbidities. This is because the patient remains conscious, airway manipulation can be avoided, and vital centres are not interfered with; hence, tachycardia, hypertension, arrhythmias, etc. can be avoided [9]. RA also helps in the early ambulation of patients. Halsted performed the first brachial plexus block [10]. Kulenkampff introduced the SCB [11]. With patients in a sitting position, he approached the brachial plexus from above the clavicle, just lateral to the subclavian artery. He injected 10 ml of 2% procaine with epinephrine after paresthesia had been achieved [12]. Real-time visualization of the anatomy and needle trajectory through ultrasound guidance enhances the safety profile and decreases procedure time avoiding any complications (inadvertent intravascular injection, neural injuries, and pneumothorax). Bupivacaine, a LA commonly used in nerve blocks, provides intraoperative anaesthesia but may be insufficient in providing postoperative analgesia. This postoperative pain is commonly treated with rescue analgesics, for example, nonsteroidal anti-inflammatory drugs and opioids. The requirement for rescue analgesics and the adverse effects of IV analgesics can be avoided by prolonging the effect of bupivacaine [13]. To avoid administering toxic doses of LAs and also to increase the efficacy of the PNBs, various adjuvants such as adrenaline [13], midazolam [13], dexamethasone [14], etc. have been added. Dexamethasone is preferred because it is more potent than hydrocortisone and it lacks mineralocorticoid activity. Including dexamethasone, steroids have very strong immunosuppressive and anti-inflammatory effects [15]. Dexamethasone is preferred as it is more potent than hydrocortisone lacking mineralocorticoid activity. Dexamethasone inhibits peripheral phospholipase, decreasing the production of cyclooxygenase and lipoxygenase [16]. It also induces vasoconstriction, prevents the release of inflammatory mediators including bradykinin (which is a major pain mediator in inflamed tissues and the operated site), and suppresses ectopic neuronal discharges [17]. Dexamethasone increases the activity of potassium channels, which prolong LA action. When lignocaine and bupivacaine are combined, they have an additive effect due to the different mechanisms of action. In our study, the most commonly used steroid, dexamethasone, was studied when given perineurally and IV in SCB under ultrasound guidance.

In our study, the mean onset of sensory block was 8.00±3.107 minutes in group P, 11.83±5.167 minutes in group I, and 15.69±4.576 minutes in group S and was found to be statistically significant between all three groups. The onset for the perineural group was eight minutes, but for the control group, it was after 15 minutes (group P was seven minutes faster). Between the perineural group and IV group, there was a difference of almost four minutes, again showing the superiority of the adjuvant in the perineural route than systemic administration. The more rapid onset of sensory block with Pd helps in relieving the patient's pain at the earliest and thus also relieving the patient's anxiety to a great extent. The findings of our study corroborated with the findings of Baloda et al. who found that patients who were injected with Pd had a faster onset and prolonged duration of sensory and motor block [18]. Our study also had findings similar to Kamel et al. who found that the onset time of sensory block was faster in the perineural group than in the systemic group [19]. In our study, we used a mixture of 7 ml of 2% lignocaine and 18 ml of 0.5% bupivacaine and found a faster onset of block with Pd, whereas Veena et al. used 25 ml of 0.5% levobupivacaine [20], Keerthana et al. used 3 mg/kg of 0.5% bupivacaine [21], and Kumar et al. used 38 ml of 0.25% bupivacaine [8] and found no significant association between the mean time for the onset of complete sensory block in IV and perineural groups. The reason for this could be that there were differences in the concentrations and dosages of the LAs used. Also, a variety of upper limb blocks were studied. The type of block may also influence the dose of LA injection.

In our study, the mean onset of motor block was 10.50±3.309 minutes in group P, 14.83±4.997 minutes in group I, and 19.29±5.727 minutes in group S, and there was a statistically significant difference. At the onset of motor block, there were a difference of nine minutes between the perineural group and control group and a difference of four minutes between the perineural group and IV group. Thus, an optimal surgical condition can be obtained faster with the perineural addition of dexamethasone when compared to IV administration. Islam et al. found that patients who were administered dexamethasone with LA had a more rapid onset of sensory and motor block and an increased duration of analgesia without any side effects than those who did not receive dexamethasone in the control group [22]. Our study findings are consistent with the results of Islam et al. proving that Pd had a faster onset in SCB and thus minimizing the wait time to initiate the surgical procedure. However, in studies conducted by Keerthana et al. (p=0.240) [21], Veena et al. (p=0.12) [20], and Kumar et al. (p=0.402) [8], the mean onset of motor blockade was comparable between both groups. This may be due to the different volumes and concentrations of the LAs in the various studies.

In this study, the mean duration of sensory block was 14.37±1.974 hours in group P, 11.60±1.694 hours in group I, and 8.48±1.765 hours in group S, and the difference was found to be statistically significant between group P and group I, group P and group S, and group I and group S. Thus, the duration of postoperative analgesia is much higher in the Pd group, enhancing faster rates of recovery in these patients. It also keeps the rebound pain phenomenon at bay for a longer period. The findings of our study are in concordance with Alarasan et al. who concluded that duration of analgesia was increased in the Pd group when compared to the placebo group, hence proving that the duration of sensory blockade was longer in Pd [23]. Albrecht et al. found that using dexamethasone with a long-acting LA increased the duration of analgesia. They also inferred that there is no difference between 4 mg and 8 mg dosages of dexamethasone [24]. Probably, further studies could be done to support or refute this finding. Kawanishi et al. concluded that Pd prolongs the duration of analgesia [25]. Results of our study highlighted a great difference in the duration of sensory block between the perineural and control groups, almost six hours longer in the perineural group. When compared to the control group, the IV group also had a greater duration of analgesia of almost three hours. Hence, we can conclude that the perineural group has a longer duration of sensory block when compared to the IV group, thus prolonging the duration of analgesia and in turn improving the recovery profile in patients who receive Pd. In spite of this, pain intensity may increase after the resolution of the nerve block effect due to increased nociceptive input after the block wears off, rebound sensitization of nerves, and increased inflammatory mediators. The management strategies for rebound pain include pre-emptive and multimodal analgesia and using a catheter-based technique to prolong the block effect. However, in the study conducted by Keerthana et al., there was no significant statistical difference between the two groups when the duration of analgesia was compared (p=0.240) [21].

In our study, the mean duration of motor block was 12.92±1.89 hours in group P, 10.30±1.622 hours in group I, and 7.58±1.57 hours in group S, and the difference among the three groups was found to have statistical significance. Pathak et al. concluded from their study that the patients who received dexamethasone with LA had a prolonged duration of block (both sensory and motor) when compared to those patients who did not receive dexamethasone [26]. With the help of this study, we can substantiate the results of our study on the duration of motor block. The perineural group showed an approximately 2.5-hour longer duration of motor block compared with the IV group and an approximately five-hour longer duration than the control group. Therefore, in comparison with the IV group, the perineural group was definitely superior in increasing the motor block. Contrary to the above findings, Abdallah et al. found that the duration of motor block was significantly prolonged in both IV and perineural groups compared with the control group (p<0.001) and in the IV group compared to the perineural group (p<0.00001) [27]. This may delay ambulation and discharge. However, for those patients who require continuous passive motion postoperatively, it may prove valuable. Hence, they concluded that IVd and Pd have similar efficacy in prolonging analgesia. In comparison with our study, the different results in this study may be due to the non-usage of adrenaline along with lignocaine and due to different methods of evaluating the block duration.

In our study, among all three groups, a total of only four patients (4.4%) (one patient each in the perineural (3.3%) and IV (3.3%) groups and two patients (6.7%) in the control group) required LA supplementation. However, statistical significance was not found. Vieira et al. found that the study group had prolonged sensory and motor block and reduced opioid usage [28]. Since they had also used clonidine apart from the study drug, the need for additional LA supplementation may not have been required.

Parthibhan et al. noticed side effects more in the IV dexamethasone group [29], while Mathew et al. noticed emesis in two patients (8%) in the perineural group in the postoperative period which was relieved with a 4 mg injection of ondansetron IV [30]. In our study, we did not find any complications in the three groups both during surgery and for the first 24 hours postoperatively. Our results found similarity with a study conducted by Kishnani et al. which also did not note any complications [31]. However, delayed complications were not recorded as the follow-up period was only for a short duration of 24 hours.

The hemodynamic parameters were comparable and showed no statistically significant differences except at 15 minutes in DBP (p=0.165; F-value-3.326) and 10 minutes in MAP (p=0.025; F-value-4.009) which were clinically not significant. Similarly, Alarasan et al. [23] and Baloda et al. [18] found no significant differences in hemodynamic parameters with Pd. However, Parthibhan et al. (p<0.05) [29] and Kishnani et al. (p<0.05) [31] found significant differences in hemodynamic parameters between the two groups at various time points.

Study limitations

ASA status III and IV patients were excluded from our study to exclude any confounding factors. Dexamethasone is known to cause an elevation in blood glucose levels by promoting gluconeogenesis, reducing glucose uptake in peripheral tissues, and increasing insulin resistance. Hence, diabetic patients were excluded from our study. Thus, the findings cannot be generalized to a significant proportion of the surgical population. Blood sugar levels were not compared between the two groups, though there were two different routes of administration of dexamethasone. Only a 24-hour follow-up of patients was done; no follow-up was conducted for the patients in the days or weeks after surgery to assess for nerve injuries or delayed infections. Furthermore, there is a lack of data on long-term functional outcomes and patient recovery, which are the ultimate goals of any analgesic intervention, both in our study and in the literature.

Dose-adjusted comparisons may provide a more precise estimate of relative efficacy. The delay in publication of the data collected during the study is a huge limitation as there may occur new evidence contradicting earlier findings.

## Conclusions

Thus, we infer that dexamethasone, when added to LAs, hastens the onset and enhances the duration of both sensory and motor blocks with perineural administration than with IV or even without any dexamethasone. Hence, we suggest that dexamethasone can be administered as an adjuvant when performing ultrasound-guided SCB for forearm and hand surgeries due to its property of speedy onset and enhanced duration of sensory and motor blockade.
